# Multicentric data analysis of the learning curve for laparoscopic Shull's repair of pelvic floor defects

**DOI:** 10.3389/fsurg.2024.1396438

**Published:** 2024-06-13

**Authors:** S. Cianci, C. Ronsini, G. Riemma, V. Palmara, P. Romeo, M. La Verde, A. S. Laganà, V. Capozzi, G. Andreoli, M. Palumbo, M. Torella

**Affiliations:** ^1^ Unit of Gynecology and Obstetrics, Policlinico “G. Martino”, Department of Human Pathology of Adult and Childhood “G. Barresi”, University of Messina, Messina, Italy; ^2^ Department of Woman, Child and General and Specialized Surgery, University of Campania “Luigi Vanvitelli”, Naples, Italy; ^3^ Unit of Obstetrics and Gynecology, “Paolo Giaccone” Hospital, Department of Health Promotion, Mother and Child Care, Internal Medicine and Medical Specialties (PROMISE), University of Palermo, Palermo, Italy; ^4^ Department of Obstetrics and Gynecology, University of Parma, Parma, Italy; ^5^ Department of General Surgery and Medical Surgical Specialties, University of Catania, Catania, Italy

**Keywords:** pelvic organs prolapse, laparoscopy, pelvic organ repair, Shull, endoscopy, gynecologic surgery, urogynecology, pelvic organs suspension

## Abstract

**Introduction:**

Pelvic organs prolapse remains a significant health concern affecting millions of women worldwide. The use of native tissues to suspend the apex has acquired relevance in urogynecologic surgery. One of the most commonly used procedures performed without mesh is the technique described by Shull, consisting of suturing the vaginal apex to the uterosacral ligaments. The objective of the study is to evaluate the learning curve of laparoscopic Shull's repair for the correction of pelvic floor defects, including the surgery time and surgical outcomes.

**Materials and methods:**

This is a retrospective study conducted at the Policlinico G. Martino, University of Messina, Messina, Italy, and Policlinico Vanvitelli, Vanvitelli University, Naples, Italy. All patients affected by grade I-IV POP, consisting of apical prolapse with or without cystocele, and who underwent laparoscopic Shull's technique for prolapse correction were enrolled. The endpoints to estimate the learning curve for the procedure were the percentage of laparoscopic procedures completed, operative time, and the early complication rate.

**Results:**

A total of 31 laparoscopic Shull repairs were collected for the study. To evaluate the learning curve of the technique, we divided the 31 cases into three different groups: Procedures 0–10; 11–20; 21–31. The parameter for evaluating technique learning was the operative time. Group 21–31 demonstrated an operative time of 97 min (SD 20), compared with 121 min (SD 23) in group 0–10 and 120 min (SD 13) in group 11–20. A comparison of these means through ANOVA showed a *p*-value of 0.01 for the entire system, and 0.95 for the comparison between 0 and 10 and 11–20, 0.04 for 0–10 vs. 21–31, and 0.02 between 11 and 20 and 21–31.

**Conclusions:**

The rate of surgical improvement in terms of time became effective after an average of 20 procedures. However, the improvement seems to be effective case by case for surgeons skilled in basic endoscopy.

## Introduction

Pelvic organs prolapse (POP) remains a significant health concern affecting millions of women worldwide, with reported prevalence rates ranging from 3% to 6% in the general female population (1). However, in parous women, the incidence increases to an average of 50% (2). Among the various treatment options available, sacrocolpopexy is a common surgical procedure for managing apical prolapse due to its durable anatomical support and low recurrence rates (2).

Laparoscopic sacrocolpopexy involves suspending the vaginal vault or apex to the sacral promontory using synthetic mesh, providing robust support and restoring normal pelvic anatomy (3). This minimally invasive approach offers several benefits, including reduced postoperative pain, shorter hospital stays, and faster recovery times (4, 5).

Despite the advantages of laparoscopic sacrocolpopexy, challenges and controversies exist, including the learning curve associated with mastering laparoscopic skills, the procedure's cost-effectiveness, and potential mesh-related complications, as indicated by the Food and Drug Administration (FDA) in 2019 to avoid the use of transvaginal mesh (6, 7). The potential severe complications associated with mesh fixation in sacrocolpopexy include infection, erosion, and chronic pain, all of which can significantly impact the patient's quality of life and may require additional surgical interventions for correction. Moreover, the success of sacrocolpopexy can be influenced by individual anatomical differences, particularly the bony structure of the pelvis. These variations can affect the proper placement and fixation of the mesh, potentially compromising the effectiveness and safety of the procedure (6, 7).

Given these considerations, the use of native tissues to suspend the apex has gained relevance in urogynecologic surgery. One commonly used procedure performed without mesh is the technique described by Shull, consisting of suturing the vaginal apex to the uterosacral ligaments, usually after hysterectomy (8). Originally performed vaginally, this technique evolved to a laparoscopic approach, allowing visualization of the ureteral route and reducing the risk of complications (4, 9). The advantages of the laparoscopic Shull procedure were demonstrated by Rardin et al. (10) in a case-control study comparing Shull's technique performed by laparoscopy after vaginal hysterectomy vs. the vaginal route alone. The authors reported better outcomes for the laparoscopic group, especially regarding the risk of ureteral damage, which was 0% in the laparoscopic group and 4% in the vaginal group. Moreover, the authors reported better outcomes for the laparoscopic group even in terms of symptomatic prolapse recurrence, demonstrating the efficacy of the laparoscopic procedure in pelvic suspension maintenance.

In this context, the capability to perform a safe procedure, reducing the complication rate, is fundamental and specific to the experience of surgeons and the number of procedures performed.

Based on this, the type of approach should be decided by considering the patient's age, clinical characteristics, and the type and grade of prolapse. Regardless of the FDA alert for the use of mesh due to the risk of rejection or infections, mesh can sometimes be a necessary device in certain conditions such as advanced stage POP, prolapse recurrence, and for patients with chronic fascial defects that cannot be resolved without synthetic tissues. Native tissue surgery could be less invasive but may sometimes be less effective. Therefore, it is essential for clinicians to carefully weigh the benefits of surgical approaches for POP and tailor treatment to individual patient characteristics and preferences.

The objective of the study is to evaluate the learning curve of laparoscopic Shull's repair for correcting pelvic floor defects, including surgical time and outcomes. The results obtained could drive the surgeons approaching this procedure to obtain better surgical performance. Moreover, as second point the surgical outcomes reported could be give more evidence regarding even the long-term efficacy of Shull procedure.

## Materials and methods

This retrospective study was conducted at the Policlinico G. Martino, University of Messina, Messina, Italy, and Policlinico Vanvitelli, Vanvitelli University, Naples, Italy. The cases analyzed were referred to the hospitals between 2021 and 2023. All patients were operated by the two surgeons who work in the two centers involved in the study. Patients affected by grade I-IV POP, including apical prolapse with or without cystocele, and who underwent laparoscopic Shull technique for prolapse correction were evaluated. The enrollment criteria included age between 18 and 80, written informed consent for the procedure, and a pre-operative complete workup. Exclusion criteria comprised previous gynecologic cancer or genital prolapse correction, autoimmune pathologies affecting pelvic stasis, contraindications to laparoscopy, previous mesh use in the pelvic area or urogynecological surgeries, previous total or subtotal hysterectomy, previous major abdominal surgery, and uteri larger more than 20 cm longitudinal and 10 cm transversal.

The pre-operative work-up included urogynecologic examination, ultrasound scan, and if required, urodynamic study and second-level radiological imaging. Data were retrieved retrospectively from databases and electronic hospital datasets. All patients were contacted post-surgery for follow-up, with the last call for all patients performed in December 2023. The surgeries were conducted by two experienced laparoscopic surgeons (more than 100 laparoscopic hysterectomies performed) but without prior experience in laparoscopic Shull technique. The endpoints to estimate the learning curve included the percentage of laparoscopic procedures completed (evaluating the conversion to laparotomy rate), operative time (from the first incision to skin closure), and early complication rate (calculated using Clavien-Dindo classification).

The surgical procedure was standardized for all patients, adhering to the technique described by Vacca et al. (11). After peritoneal cavity access and positioning of three suprapubic 5 mm trocars, the procedure began with round ligament section, followed by development of retroperitoneal spaces identifying the ureteral pelvic course, para-rectal, and para-vesical spaces. The uterine artery was closed at the origin using a surgical clip, and if bilateral salpingo-oophorectomy was necessary, the ovarian pedicles were cut. The Okabayashi space was developed, and the pelvic tract of the ureter was isolated while maintaining ureteral vascularization. Subsequently, the corporal branches of uterine arteries were isolated and cut at the level of the uterine isthmus. The vesicovaginal septum was developed, and the posterior part of the pubocervical fascia was dissected up to the vagina. Hysterectomy was then completed through colpotomy with a monopolar hook, and the specimen was removed through the vagina. Vaginal apex suspension was performed by identifying three points of the vagina from lateral to medial. Three suspension sutures per side were applied and tied to close the vagina and connect the vaginal points, pubocervical fascia, and uterosacral ligaments to suspend the vagina. Stitches were closed using laparoscopic intra-corporeal knots, no barbed sutures were used. If needed, vaginal cystopexy was performed after the laparoscopic step. The uterine manipulator were used at the surgeon's discretion. For all cases a multifunction instrument was routinary used. The catheter was removed on day 1, and routine blood tests were performed on the same day.

### Statistical analysis

For categorical variables, results were presented as absolute numbers and percentages, and for continuous variables, as mean ± standard deviation (SD). Pearson's chi-square or Fisher's exact test was used to assess categorical variables. Tukey's honestly significant difference (HSD) was employed in pairwise comparison after one-way analysis of variance (ANOVA) to examine differences between means. The number of procedures performed by operators and the length of the surgical procedure were correlated using linear regression analysis to evaluate the surgeons' learning curve. STATA 14.1 (StataCorp LLC, College Station, TX, USA) was used for all statistical analysis and plots.

## Results

A total of 31 laparoscopic Shull repairs were collected for the study. All procedures performed during the specified period, meeting the inclusion criteria, were enrolled. In 84% of cases, hysterectomy was associated with cystopexy techniques for concurrent cystoceles.

To avoid operative time bias related to additional surgical procedures, we conducted a logarithmic regression to evaluate the effect of cystopexy on operative time in the three patient groups. The independent variable, cystopexy, did not show a correlation with the outcome of operative time (Table 1).

**Table 1:** Logarithmic regression of cystopexy on operative time.

	0–10 operation time			11–20 operation time			21–31 operation time		
Characteristic	Beta	95% CI^a^	*p*-value	Beta	95% CI^a^	*p*-value	Beta	95% CI^a^	*p*-value
Cystopexy	12.3	−3.3 17.1	0.2						
				11.8	−2.4 22.7	0.3			
							9.9	−4.14 6.8	0.5

^a^ CI, confidence interval.

In 6% of cases, a rectocele correction technique was also associated. The mean age of the enrolled patients was 61 years, and the mean BMI was 28. The 97% of the enrolled patients were postmenopausal, and 45% had undergone previous surgeries. All data regarding the preoperative characteristics of the sample are summarized in Table 2.

**Table 2:** Baseline characteristics of included patients and procedures.

Characteristics	Mean (SD) or *N* (%)
Age	61 (7)
BMI	28 (3)
Menopausal status	30 (97)
previous surgery	14 (45)
Hysterocele (Grade)	
1	6 (19)
2	15 (48)
3	8 (26)
4	2 (6)
Cystocele (Grade)	
1	6 (19)
2	15 (48)
3	5 (16)
Rectocele (Grade)	
1	2 (6)
Stamey score (Grade)	
1	8 (26)
2	14 (45)
3	9 (29)
PC test	
0	3 (10)
1	13 (42)
2	6 (19)
3	0 (0)
n/a	9 (29)
*Q* tip test	
<30°	4 (13)
30°–60°	13 (42)
>60°	5 (16)
n/a	9 (29)

### Surgical outcomes

The entire procedure was completed by laparoscopy. No conversion to laparotomy was recorded. To simplify the evaluation of the learning curve of the technique, we divided the 31 cases into 3 different groups. Procedures 0–10; 11–20; 21–31. The parameter for evaluating technique learning was the operative time. Group 21–31 demonstrated an operative time of 97 min (SD 20), compared with 120 min (SD 23) in group 0–10 and 118 min (SD 13) in group 11–20. A comparison of these means through Anova showed a *p* value of 0.01 for the whole system and 0.96 for the comparison between 0 and 10 and 11–20 and 0.04 for 0–10 vs. 21–31 and 0.02 between 11 and 20 and 21–31. This data is summarized in Table 3.

**Table 3:** Mean procedure time according to number of procedures (ANOVA with tukey HSD).

Procedure no.	Procedure time. Mean (SD)	*p*-value
0–10	121 (23)	0.01
11–20	120 (13)	
21–31	97 (20)	

Tukey HSD post-estimations: 0–10 vs. 11–20 = 0.96; 0–10 vs. 21–31 = 0.04; 11–20 vs. 21–31 = 0.02.

**Figure 1:**
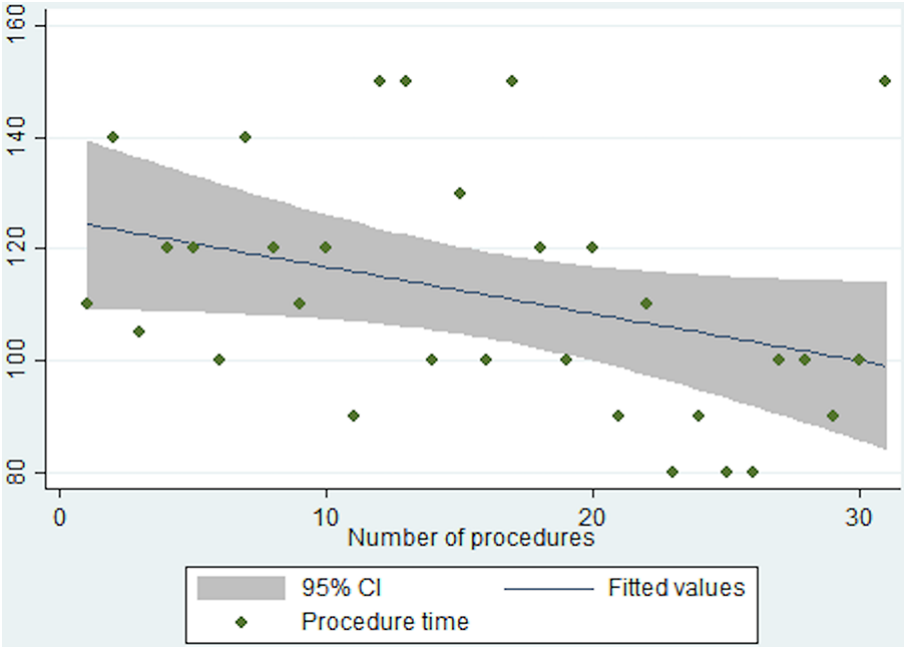
Linear regression of single procedures’.

A linear regression of single procedures’ data showed a progressive decrease of surgical time, case-by-case. (Figure 1) r2: 0.1; coeff. −0.8 (95% CI: −1.7 to −0.1); SE 0.4; *p* = 0.05.

Moreover, to evaluate the efficacy and safety of the technique, we assessed complication rates according to Clavien-Dindo's classification (12). Overall, 10 complications occurred (32%), with only 1 classified as grade 3 (pulmonary embolism) (3%).

Regarding efficacy, Shull's technique demonstrated a statistically significant decrease in the rates of stress incontinence (71% before surgery vs. 3% after surgery), bulking symptoms (58% vs. 0%), and incomplete voiding (42% vs. 0%) (*p* < 0.01). Urge incontinence, nicturia, urinary hesitation, and constipation showed a decreasing trend after surgery, although not reaching statistical significance.

In our case series, only 2 patients (6%) experienced recurrence of prolapse within 6 months after surgery. All surgical outcomes are summarized in Table 4.

**Table 4:** Clinical and surgical outcomes.

Symptoms	Before surgery, *n* (%)	After surgery, *n* (%)	*p*-value
Urge incontinency	4 (13)	1 (3)	0.35
Stress incontinency	22 (71)	1 (3)	<0.01
Nicturia	5 (16)	1 (3)	0.19
Bulking symptoms	18 (58)	0 (0)	<0.01
Urinary hesitation	4 (13)	2 (6)	0.67
Incomplete voiding	13 (42)	0 (0)	<0.01
Constipation	13 (42)	10 (32)	0.60
Recurrence	/	2 (6)	/
Complications	/	5 (16)	/
Clavien-Dindo 1		3 (10)	
Clavien-Dindo 2		1 (3)	
Clavien-Dindo 3		1 (3)	
Fever		2 (6)	
Anemia		1 (3)	
UTI		1 (3)	
Pulmonary embolism		1 (3)	

## Discussion

The vaginal uterosacral ligament suspension described by Shull et al. (8) for POP repair is one of the most used surgical procedures adopted without the use of mesh. Following the FDA's advice regarding synthetic devices, the procedure gained more prominence. However, the difficulty in recognizing the uterosacral ligament through the vagina and the risk of ureteral damage associated with the blind procedure led to modifications in the technique, shifting toward laparoscopy. The advantages of laparoscopy for urogynecologic surgery have been demonstrated by various studies (13), including a randomized study by Carey et al. (14). These advantages include the ability to visualize and dissect all anatomic structures, minimizing blood loss and the risks of complications, primarily associated with blind procedures (15, 16).

Apart from the surgical techniques, that could be very different accounting hundreds of different techniques the aim of treatment should always be to obtain the best results while minimizing the risk to patients to ensure a good quality of life (17, 18). This aspect has been investigated in the literature, and the available evidence demonstrates that vaginal native tissue repair for pelvic organ prolapse is effective from a surgical standpoint. Additionally, it has a positive impact on quality of life and sexual function, as shown in the study by Schiavi et al. (19).

For menopausal women, the most commonly adopted procedure is uterosacral vaginal suspension after total hysterectomy, which was also performed in our study prior to vaginal suspension. The usefulness of hysterectomy was previously investigated by Rosen et al. (20), who compared two groups of patients affected by POP undergoing pelvic repair with uterosacral suspension, with one group undergoing hysterectomy and the other not. The results showed no significant differences in surgical outcomes between the hysterectomy and non-hysterectomy groups. However, it was noted that the risk of cervix elongation requiring reintervention was associated with patients who retained their uterus.

The choice of surgical procedure is not completely standardized and is subjective, depending on the center or the surgeon's experience. However, in a study by Campagna et al. (17) comparing laparoscopic high uterosacral ligament suspension vs. laparoscopic sacral colpopexy, both approaches were deemed safe and feasible, although laparoscopic sacral colpopexy was found to be more effective for multicompartment prolapse or severe (grade 3–4) prolapse. Therefore, the authors suggested that high uterosacral suspension should be reserved for patients with grade 1–2 prolapse.

Based on available literature, ureteral damage represents the most common complication during Shull procedures, with an average incidence of about 11% for vaginal route procedures (4). This data was confirmed in a recent meta-analysis, which reported better outcomes for laparoscopic procedures (21).

Based on our results, laparoscopic Shull repair appears to be effective in terms of surgical outcomes, as we recorded only one major complication (grade III), and in terms of pelvic floor correction effects, as we observed statistically significant results, especially regarding the resolution of stress urinary incontinence, bulking symptoms, and incomplete voiding (*p* < 0.05).

Regarding the learning curve, two points seem to be particularly relevant. Firstly, it appears that the learning progression begins from the first procedure and improves continuously. However, our data demonstrate that after 20 procedures, the training curve could be considered concluded. In fact, as reported in Table 3, the time gap between 0 and 10 and 11–20 procedures is not significant. However, after 20 procedures, in the 21–31 procedure group, the time is significantly reduced. Moreover, comparing our data with available literature (11, 22), which reports an average time of 80 min (range 70–120 min), after 20 procedures, the operative time falls within the reported range. Our surgical time is likely even lower if we consider that some procedures included cystopexy.

The strength of our study lies in the fact that, to our knowledge, this is the first study in the literature focused on the learning curve of this specific procedure, and the fact that the procedure was standardized in all selected cases. This study could be useful for surgeons approaching this type of surgery, providing insights on how they can manage their training and determine when they are ready to perform the procedure independently. The limitation of the study is related to its retrospective nature and the short follow-up period.

Moreover, even though we obtained significant results, a higher number of patients should be enrolled to gather more concrete data, and even surgeons with intermediate experience could be tested. In the present study all procedures were performed by skilled surgeons in basic laparoscopy. However, in our opinion, this procedure cannot be approached by beginner laparoscopic surgeons as it requires skills in dissection and knotting.

In conclusion, laparoscopic Shull repair appears to be feasible and effective in terms of surgical and urological outcomes. The learning curve to perform the procedure easily is about 20 cases for surgeons skilled in basic laparoscopy.

## Data Availability

The raw data supporting the conclusions of this article will be made available by the authors, without undue reservation.
